# Bunched and Madm Function Downstream of Tuberous Sclerosis Complex to Regulate the Growth of Intestinal Stem Cells in Drosophila

**DOI:** 10.1007/s12015-015-9617-5

**Published:** 2015-09-02

**Authors:** Yingchao Nie, Qi Li, Alla Amcheslavsky, Juan Carlos Duhart, Alexey Veraksa, Hugo Stocker, Laurel A. Raftery, Y. Tony Ip

**Affiliations:** Program in Molecular Medicine, University of Massachusetts Medical School, Worcester, MA 01605 USA; Department of Biology, University of Massachusetts Boston, Boston, MA 02125 USA; Institute of Molecular Systems Biology, ETH Zürich, Zürich, Switzerland; School of Life Sciences, University of Nevada Las Vegas, Las Vegas, NV 89154 USA

**Keywords:** Bunched, Drosophila, Intestine, Madm, Stem cells, TSC-22, Tuberous sclerosis complex

## Abstract

The Drosophila adult midgut contains intestinal stem cells that support homeostasis and repair. We show here that the leucine zipper protein Bunched and the adaptor protein Madm are novel regulators of intestinal stem cells. MARCM mutant clonal analysis and cell type specific RNAi revealed that Bunched and Madm were required within intestinal stem cells for proliferation. Transgenic expression of a tagged Bunched showed a cytoplasmic localization in midgut precursors, and the addition of a nuclear localization signal to Bunched reduced its function to cooperate with Madm to increase intestinal stem cell proliferation. Furthermore, the elevated cell growth and 4EBP phosphorylation phenotypes induced by loss of Tuberous Sclerosis Complex or overexpression of Rheb were suppressed by the loss of Bunched or Madm. Therefore, while the mammalian homolog of Bunched, TSC-22, is able to regulate transcription and suppress cancer cell proliferation, our data suggest the model that Bunched and Madm functionally interact with the TOR pathway in the cytoplasm to regulate the growth and subsequent division of intestinal stem cells.

**Electronic supplementary material:**

The online version of this article (doi:10.1007/s12015-015-9617-5) contains supplementary material, which is available to authorized users.

## Introduction

Homeostasis and regeneration of an adult tissue is normally supported by resident stem cells. Elucidation of the mechanisms that regulate stem cell-mediated homeostasis is important for the development of therapeutics for various diseases [1].

The intestine with fast cell turnover rate supported by actively proliferating stem cells is a robust system to study tissue homeostasis [2]. In the mouse intestine, two inter-converting intestinal stem cell (ISC) populations marked by Bmi1 and Lgr5 located near the crypt base can replenish cells of various lineages along the crypt-villus axis [3–5]. Furthermore, recent data suggest that Lgr5+ cells are the main stem cell population and that immediate progeny destined for the secretory lineage can revert to Lgr5+ stem cells under certain conditions [6, 7]. Together, the results suggest previously unexpected plasticity in stem cell maintenance and differentiation in the adult mammalian intestine.

In the adult *Drosophila* midgut, which is equivalent to the mammalian stomach and small intestine, ISCs are distributed evenly along the basal side of the monolayered epithelium to support repair [8–11]. The maintenance and regulation of Drosophila midgut ISCs depend on both intrinsic and extrinsic factors. When a midgut ISC divides, it generates a renewed ISC and an enteroblast (EB) that ceases to divide and starts to differentiate. The ISC-EB asymmetry is established by the Delta-Notch signaling, with Delta in the renewed ISC activating Notch signaling in the newly formed neighboring EB [11–13] (see Fig. S1A). Growth factors such as Wingless/Wnt, insulin-like peptides, Decapentaplegic/BMP, Hedgehog and ligands for the EGF receptor and JAK-STAT pathways are secreted from surrounding cells and constitute the niche signals that regulate both ISC division and EB differentiation [14–20]. ISC-intrinsic factors including Myc, Target of Rapamycin (TOR) and Tuberous Sclerosis Complex act to coordinate the growth and division of ISCs [21–23]. Furthermore, chromatin modifiers such as Osa, Brahma and Scrawny function within ISCs to regulate Delta expression or ISC proliferation [24–26].

Here we report the identification of the leucine zipper protein Bunched (Bun) and the adaptor protein myeloid leukemia factor 1 adaptor molecule (Madm) as intrinsic factors for ISC proliferation. A single *bun* genomic locus generates multiple predicted transcripts that encode 4 long isoforms, BunA, F, G and P, and 5 short isoforms, BunB, C, D, E, H and O [27–29]. The first identified mammalian homolog of Bun is TGF-β1 stimulated clone-22 (TSC-22). In the mouse genome four different *TSC-22* domain genes also encode multiple short and long isoforms [30–33]. All isoforms of Bun and TSC-22 contain an approximately 200 amino acids C-terminal domain where the conserved TSC-box and leucine zippers are located (Fig. S1E). The originally identified TSC-22 is a short isoform and various assays suggest that it suppresses cancer cell proliferation and may function as a transcriptional regulator [32–35]. Meanwhile, in Drosophila, the long Bun isoforms positively regulate growth, while the short isoforms may antagonize the function of long isoforms [27, 28]. Transgenic fly assays also demonstrate that the long TSC-22 can rescue the *bun* mutant phenotypes, whereas short isoforms cannot [36]. These results suggest an alternative model that the long Bun isoforms positively regulate proliferation, while the short isoforms may dimerize with and inhibit the functions of long isoforms [27, 28, 36].

Madm also can promote growth. The long isoform BunA binds to Madm via a conserved motif located in the N-terminus that is not present in the short Bun isoforms [36] (Fig. S1E, F). The molecular function of this novel BunA-Madm complex, nonetheless, remains to be elucidated. Our results in this report demonstrate that Bun and Madm modulate the Tuberous Sclerosis Complex-target of Rapamycin (TOR)-eIF4E binding protein (4EBP) pathway to regulate the growth and division of ISCs in the adult midgut.

## Materials & Methods

### Drosophila Stocks

Fly stocks were maintained at room temperature (approximately 22 °C) in yeast extract/cornmeal/molasses/agar food medium. *w*^*1118*^ was used as wild type control to cross with esg^ts^ > GFP in different experiments. Transgenic RNAi fly stocks used were: *bun* RNAi1 (VDRC19679), *bun* RNAi2 (VDRC19680), *Madm* RNAi1 (VDRC27346), *Madm* RNAi2 (VDRC27347), *hpo* (VDRC104169, TRiP27661), *msn* (TRiP28791), *TSC2* (VDRC6313). Transgenic fly stocks UAS-InR^A1325D^, UAS-EGFR^A887T^, UAS-Notch^DN^, UAS-Vein, UAS-Upd3 and UAS-Upd has been previously described [8, 9, 11, 17]. UAS-Rheb is from Bloomington (9689). The *Madm* fly stocks *FRT82BMadm*^*3T4*^, *FRT82BMadm*^*2D2*^ were previously described [36]. The *bun* and *Madm* fly stocks UAS-BunA, UAS-BunB, UAS-Madm, UAS-Madm;UAS-BunA, FRT40A*ΔGE12921*, FRT40A*bun*^*200B*^, FRT40*ΔGE12921*;UAS-Ras^V12^, FRT40A*bun*^*200B*^;UAS-Ras^V12^ were previously described [27, 28, 36]. UAS-Ras^v12^ on III was a gift from Nam Moon.

### MARCM Clonal Analysis

Fly stocks were crossed to generate hsFLP, tubGal4, UAS-GFP; FRT82B *Madm*^*2D2*^/FRT82B tubGal80 and hsFLP, CD8GFP; FRT40A *bun*^*200B*^/FRT40A tubGal80; tubGal4 and hsFLP, CD8GFP; FRT40A *bun*^*200B*^/FRT40A tubGal80; UAS-Ras^V12^/tubGal4. The final crosses and progenies were kept at room temperature. Flies of 5–7 day old were heat shocked twice in a single day at 37 °C for 1 h each time. Flies were then kept at room temperature for 7 days for FRT82B alleles and at 18 °C for 7 days for FRT40A alleles before dissection.

### Transgenic SBP-BunA and BunA-NLS Constructs

For UAS-BunA-SBP fusion tagged at the C-terminus, BunA was PCR amplified with forward primer (BunAcFor primer) 5′-GCGGAATTCCCAAATAATCCAAGAATCGTTAGC and reverse primer (BunAcRev primer) 5′- GCGTCTAGAGGACATGGGACCATTGGCTGT and cloned into pUAST-attB-cSBP, using the restriction sites as underlined. For UAS-SBP-BunA fusion tagged at the N-terminus, BunA was amplified with BunAnFor primer 5′-GCGGAATTCAGCCGAGAATCAAAGTGC and BunAnRev primer 5′-GCGTCTAGAAGAAAGAGACTGCGGCTGAG and cloned into pUAST-attB-nSBP. For SBP-tagged BunA-NLS, a forward primer 5′-GCGGAATTCA**GATCCAAAAAAGAAGAGAAAGGTA**GCCGAGAATCAAAGTGC and the BunAnRev primer as above were used. Similarly, the BunAcFor primer as above and a reverse primer: 5′-GCGTCTAGA**TACCTTTCTCTTCTTTTTTGGATCTACCTTTCTCTTCTTTTTTGGATC** GGACATGGGACCATTGGCTGT were used on the pUAST-BunA as a PCR template to generate 2XNLS. Underlined sequence in bold is SV40 large T antigen nuclear localization signal. Amplified PCR products were cloned into pUAST-attB-nSBP and pUAST-attB-cSBP, respectively, resulting plasmids pUAST-attB-SBP-NLS-BunA and pUAST-attB-BunA2xNLS-SBP. Both plasmids were then digested with HindIII and StuI, and fused together to make pUAST-attB-SBP-NLS-BunA-2xNLS-SBP, in short as SBP-BunA-NLS in Fig. 3. Transgenic flies were generated in *w*^*1118*^ background using phiC31-mediated insertion (Rainbow Transgenic Flies, Inc. Camarillo, CA). UAS-BunA-SBP was inserted using the strain y[1] M{vas-int.Dm}ZH-2A w[*]; M{3xP3-RFP.attP}ZH-86Fb and UAS-SBP-BunA and UAS-SBP-BunA-NLS were inserted using the strain R8622-modified 8622 (y[1] w[67c23]; P{y[+t7.7] = CaryP}attP2).The transgenic SBP-Delta (Dl) construct contained a SBP sequence inserted into an approximately 85 kb of genomic *Delta* promoter and coding sequence and generated a fusion at the cytoplasmic portion of Delta. The attP2 stock was used for this transgenic insertion. The non-tagged version BunA-nls was generated independently in the UAST vector and introduced into transgenic flies by random *P* element-mediated transformation. The nls sequence and location are listed in Fig. S3.

### Feeding Experiments

Feeding experiments were performed as previously described [9]. Approximately 50 flies aged for 5–7 days were kept in vials with regular food for 2 days at 29 °C to enable the dsRNA expression. Feeding was performed subsequently by dividing the flies into vials with either Whatman paper soaked with 5 % sucrose or plus 5 % DSS. Flies were fed at this condition at 29 °C for another 2 days before dissection for immunostaining and p-H3 counts.

### Immunofluorescence Confocal Microscopy

Female flies were dissected for analysis routinely because of the bigger size. Fly head was removed and the gastrointestinal tract was pulled from the posterior end directly into 4 % formaldehyde in PBS (Mallinckrodt Chemicals) for fixation up to 3 h. For Delta staining, guts were fixed only for 1 h. Immunostaining procedure has been described previously [9]. Briefly, guts were permeabilized with 0.1 % Triton X-100/PBS, followed by blocking in 5 % normal horse serum, 0.5 % BSA, 0.1 % Triton X-100, 1XPBS for 1.5 h. Primary antibody was diluted in blocking buffer and incubated with guts at 4 °C overnight. Anti-sera used were: anti-Delta (mouse monoclonal, 1:100 dilution, DSHB, Iowa), anti-Prospero (mouse monoclonal, 1:100, DSHB), anti β-galactosidase (rabbit polyclonal, 1:50,000, Cappel, MP Biomedicals), anti-pH3 (rabbit polyclonal, 1:1000, Millipore), anti-SBP (mouse monoclonal, 1:100, Santa Cruz), anti-p-4EBP (Thr37/46) (rabbit polyclonal 1:100, Cell signaling). Subsequent wash and secondary antibody incubation were in 0.5 % BSA, 0.1 % Triton X-100, 1XPBS. Secondary antibody used were goat anti-mouse IgG conjugated to Alexa 568 or Alexa 633 (1:1500, Molecular Probes/Invitrogen), or goat anti-rabbit IgG conjugated to Alexa 555 or Alexa 633 (1:2000, Molecular Probes/Invitrogen). After 3 washes in 0.1 % Triton X-100, 1XPBS, guts were mounted in a solution of 1:1 ratio of 1XPBS:Vectorshield with DAPI (Vector Lab) for microscopy analysis. Images were taken by Nikon Spinning Disk Confocal microscope (UMass Medical School Imaging Core Facility). The acquisition and processing software is MetaMorph (Molecular Devices) and single optical sections are shown as images in the figures.

## Results

### Bun and Madm are Required in ISC for Proliferation

**Fig. 1:**
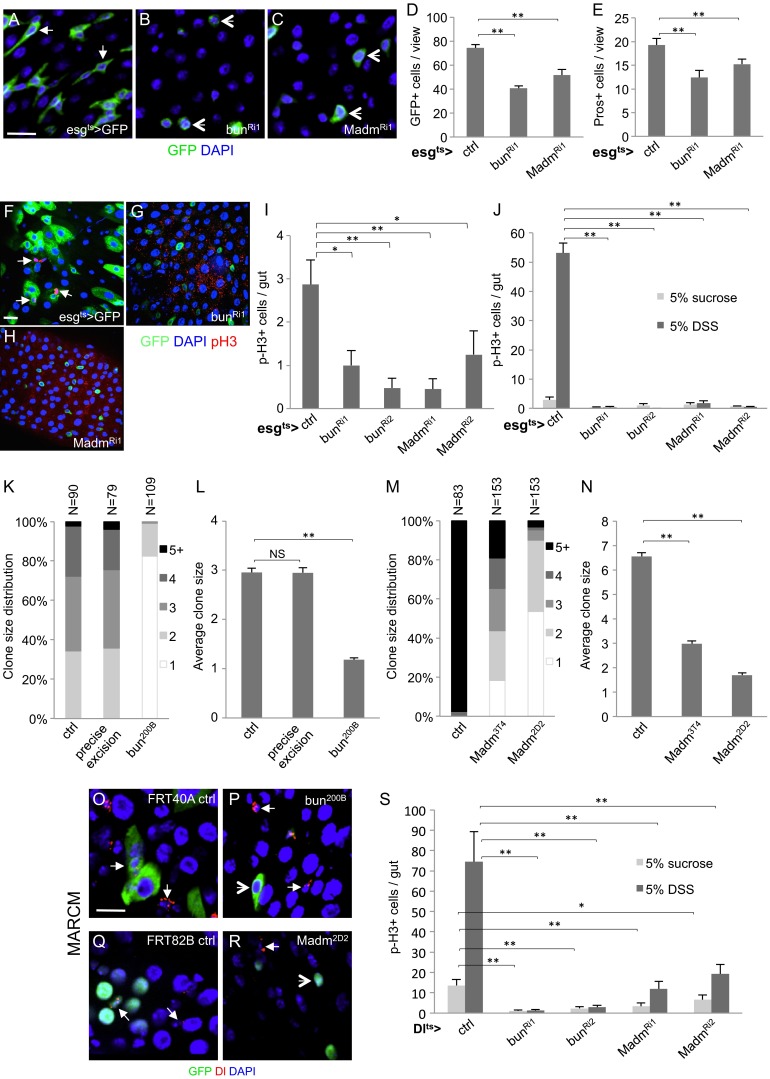
Bun and Madm are required in ISCs for proliferation. **a-c** Confocal images of midguts from adult flies that had the esg^ts^ > driving the UAS-GFP as control, or the UAS-bun^RNAi^ or UAS-Madm^RNAi^ as indicated. Flies were kept at room temperature after eclosion for 5–7 days and then shifted to 29 °C for 4 days to induce the dsRNA expression. The arrows in A indicate 2 examples of normal GFP+ precursor cells, and the arrowheads in B and C indicate examples of defective GFP+ cells after RNAi. DAPI staining for DNA is blue. The scale bar is 20 μm and all images were processed in the same way. **d-e** Quantification of GFP+ and Pros + cells were carried out by counting the positive cells in 40X objective confocal images of midguts from each genotype. The area per view was approximately 0.08 mm^2^. 7 to 12 images of each genotype were counted and the average was plotted. The *error bar* represents standard error of the means. All P values are from Student’s *t* test, and ** is *P* < 0.01. **f-j** The flies were aged and temperature shifted the same way as described above. In F-H, representative confocal images are shown for GFP and phospho-histone 3 (p-H3) staining. In I-J, the flies were split into parallel vials with sucrose solution or with sucrose + 5%DSS. Fly guts were dissected and stained for the p-H3 mitotic marker and positive staining was counted in the whole gut. More than 10 guts from each experimental condition were counted and the average was plotted. * is *P* < 0.05. **k-n** MARCM clonal analyses in midgut ISCs of *bun* and *Madm* mutants. Eclosed flies were aged at room temperature for 5–7 days and MARCM clones were generated after 37 °C heat shock of the flies to induce FLP expression and mitotic recombination. The flies were returned to 18 °C (*bun* mutants) or room temperature (*Madm* mutants) for 7 days and midguts were dissected for analysis. The FRT40A wild type chromosome (control) and the FRT40A-*bun* ^*ΔGE1292*^ (precise excision) chromosome were used as controls for *bun* mutants. The FRT82B wild type chromosome was used as the control for *Madm* mutants. N is the number of clones counted for each genotype. The clone size distribution and average clone size were plotted. NS is non-significant with *P* > 0.05. **o-r** Confocal images containing MARCM clones in midguts of control and mutant flies. The UAS-GFP used in the FRT40A experiment was cytoplasmic and in the FRT82B experiment was nuclear, all labeling the MARCM clones. The arrows in all these panels indicate examples of Delta (Dl) + cells. The arrowheads indicate examples of mutant cells. **s** Mitotic cell counts in adult midguts using the ISC-specific *Delta(Dl)* ^*ts*^ driver. The control flies were Dl^ts^ > GFP and two different *bun* ^*RNAi*^ and *Madm* ^*RNAi*^ lines were examined. Cells positively stained for p-H3 were counted for whole guts and the average was plotted

We used the *escargot* promoter-Gal4 (esg>) that expresses in both ISCs and EBs (Fig. S1A) [11] to perform RNA interference (RNAi) assays after crossing with UAS-directed transgenes expressing double stranded RNAs. The tubulin-Gal80^ts^ and UAS-mCD8GFP were included (together abbreviated as esg^ts^ > GFP) where the Gal80^ts^ temperature sensitive repressor provided temporal control of the Gal4 activity and the GFP expression helped to visualize the cells that expressed the dsRNAs. Two transgenic dsRNA fly lines each for a *bun* or a *Madm* construct showed similar defects within the adult midgut. The RNAi knockdown efficiency for *bun* was examined by immunofluorescent staining of the SBP tagged version of Bun protein (Fig. S2A-G), and quantification of *Madm* RNAi by qPCR also showed substantial knockdown of the targets (Fig. S2H). Quantification of GFP+ cells, which represent both ISCs and EBs under the esgGal4 driver, using confocal images of the posterior midguts showed a significant reduction after *bun*^*RNAi*^ or *Madm*^*RNAi*^ (Fig. 1a-d). The morphology of the remaining GFP+ cells after RNAi was rounded. Comparing to normal precursor cells that should show more extended cell shape (arrows in Fig. 1a) or increased cell size at later stage, the precursor cells after *bun*^*RNAi*^ or *Madm*^*RNAi*^ were not differentiating (arrowheads in Fig. 1b, c). Prospero (Pros) is expressed in all enteroendocrine cells (EEs) [11], and quantification of Pros + cells also showed reduced number of EEs after the RNAi (Fig. 1e).

ISCs are the only mitotic cells in the adult midgut, and ISC division is critical for the maintenance of midgut epithelial cell population [10, 11]. The mitotic marker phosphorylated histone 3 (p-H3) staining reveals condensed chromatin of ISCs at the M phase [10, 11]. Quantification of p-H3 staining showed that the number of mitotic cells was much reduced after *bun*^*RNAi*^ or *Madm*^*RNAi*^ (Fig. 1f-i). The relatively young flies of 5–7 days after eclosion used as controls for these experiments normally had low number of mitotic ISCs captured by the p-H3 staining in fixed midguts. Therefore, we fed the flies with the tissue damaging agent dextran sulfate sodium (DSS) to increase the number of mitotic ISCs in the midguts [9]. After this challenge, the *bun*^*RNAi*^ or *Madm*^*RNAi*^ midguts still showed very low p-H3 count (Fig. 1j), demonstrating that each of these two genes in precursor cells was critical for ISC division during normal homeostasis and tissue damage response.

To gain insight into the cell type requirement of *bun* and *Madm*, we used loss of function alleles of the two genes to perform clonal analyses by the Mosaic Analysis of Repressible Cell Marker (MARCM) technique. This technique is based on FRT-mediated mitotic recombination to randomly generate a small number of homozygous mutant cells or clones that should include an ISC and its progenies all positively marked by GFP [9, 11]. The *P* element excision induced deletion *bun*^*200B*^ is a null mutant [28] and ISC mutant clones for this allele marked by GFP expression remained as single cells. In comparison, the cell numbers of control clones were higher over the same experimental period when using wild type flies or a precise excision allele (*bun*^*ΔGE1292*^) isolated in parallel with the *bun*^*200B*^ deletion (Fig. 1k, l, o, p). Similar experiments using two different alleles of *Madm* revealed a reduction of cell number in the mutant clones when compared to the control clones (Fig. 1m, n, q, r). *Madm*^*3T4*^ is a hypomorphic allele and *Madm*^*2D2*^ is likely a null allele based on the nature of the mutation and the tissue growth assays in developing eye imaginal discs [36]. The MARCM clone size reduction in the midgut of these *Madm* alleles matched their previously described phenotypic strength in eye discs. The confocal images revealed the GFP+ single cells of the *bun* and *Madm* mutant alleles (arrowheads in Fig. 1p, r).

We analyzed further the ISC-specific requirement in the adult midgut by using the Delta^ts^ > driver, which has a more specific albeit weaker expression in ISCs than that of the esg^ts^ > driver [37]. The number of mitotic cells in the midgut represented by p-H3 staining was substantially reduced after *bun*^*RNAi*^ or *Madm*^*RNAi*^, especially when compared with the DSS fed control flies (Fig. 1s). Together with the MARCM results, we conclude that most of the loss of midgut proliferation phenotype is due to the requirement of *bun* and *Madm* within the ISCs.

### Bun and Madm Function Downstream of the Major Niche Signaling Pathways

**Fig. 2:**
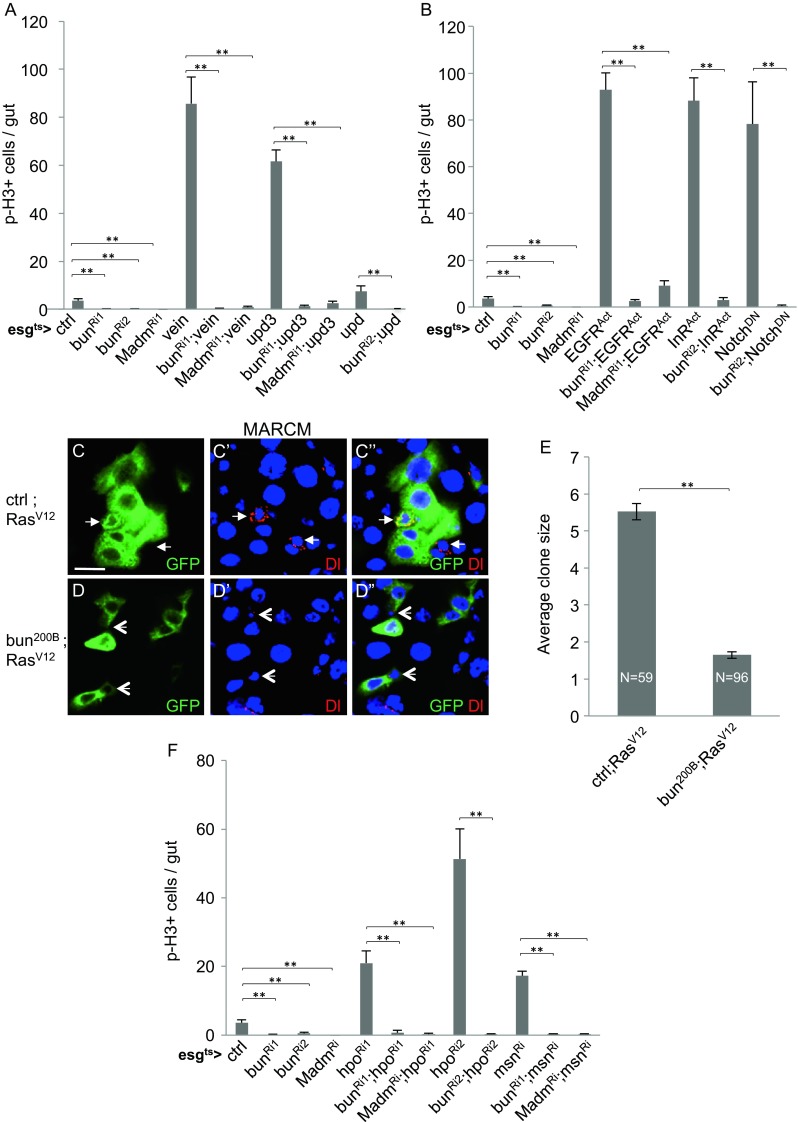
Bun and Madm function independently of the major niche signaling pathways. **a-b** Transgenic constructs as indicated were crossed together with the esg^ts^ > driver. The flies were aged and shifted to 29 °C as described in Fig. 1. Staining and quantification of p-H3 positive cells were performed as described above. Transgenes located on X (*bun* ^*RNAi2*^) or 2^nd^ (*bun* ^*RNAi1*^, *Madm* ^*RNAi1*^ and *Madm* ^*RNAi2*^) chromosome were used depending on the chromosomal location of the other transgenes used for the experiments. **c-e** MARCM clonal analysis of *bun* mutant and gain of function Ras^v12^. The control was FRT40A-*bun* ^*ΔGE1292*^ precise excision crossed with UAS-Ras^v12^. The mutant was FRT40A-*bun* ^*200B*^ crossed with UAS-Ras^v12^. MARCM clones were induced as described in Fig. 1. The arrows in C indicate Delta + ISCs. The arrowheads in D indicate *bun* mutant cells. The GFP+ cell numbers in control and mutant *bun* clones were plotted as average in E. **f** Experimental conditions were the same as described in panels A and B, except that the UAS-*hpo* ^*RNAi*^ or UAS-*msn* ^*RNAi*^ was used to increase mitotic cell count in midguts

Recent reports have demonstrated that multiple conserved signaling pathways regulate ISC proliferation. We investigated whether Bun and Madm may be a component of one of these regulatory pathways. The EGFR and JAK-STAT pathways are the major growth regulatory pathways in the midgut [14]. Vein and Upd3 are ligands of these two pathways, respectively, and overexpression of Vein or Upd3 by using the esg^ts^ > is sufficient to induce midgut hyperplasia by promoting both ISC division and EB differentiation, concomitant with highly increased p-H3 counts. The inclusion of the *bun*^*RNAi*^ or *Madm*^*RNAi*^ constructs efficiently suppressed this proliferation phenotype (Fig. 2a). Other Upds have also been implicated in midgut homeostasis [38]. Therefore, we also performed Upd overexpression, which caused a much milder midgut proliferation. It is nonetheless similarly suppressed by the loss of Bun or Madm (Fig. 2a).

The overexpression of a constitutively active EGFR induced high proliferation, which was similarly suppressed by *bun*^*RNAi*^ or *Madm*^*RNAi*^ (Fig. 2b). The activation of the EGF pathway can be mimicked by expression of the gain of function Ras^V12^. Control MARCM clones that expressed Ras^V12^ had significantly increased cell number, which was suppressed when the *bun*^*200B*^ mutant was also present in the clones (Fig. 2c, d, e). All these results demonstrate that *bun* and *Madm* loss of function can suppress the over-proliferation phenotype induced by gain of function EGFR and JAK-STAT pathway components.

We also tested the gain of function insulin receptor that we previously showed to function in ISC to promote proliferation [9]. This activation was also largely suppressed by *bun*^*RNAi*^ (Fig. 2b). Meanwhile, the Notch pathway establishes the ISC-EB asymmetry by restricting Delta expression [13]. The Notch dominant negative construct expressed in the precursor cells would lead to the formation of more ISCs after each division, thereby increasing the p-H3 count substantially. Combining the *bun*^*RNAi*^ with the Notch dominant negative construct resulted in very low proliferation (Fig. 2b). Meanwhile, the normal ISC-EB asymmetry probably was not disrupted after *bun*^*RNAi*^ or *Madm*^*RNAi*^, because we frequently observed only one of the cells in the cell nests exhibited the Su(H)lacZ expression, representing normal Notch pathway activation (Fig. S1B-D).

Two other pathways that can increase ISC proliferation are the Hippo and Misshapen, both of which are Sterile 20/germinal center kinases [39–43]. RNAi-mediated knockdown of either kinase led to increased ISC proliferation, possibly via the relief of the transcriptional co-activator Yorkie to stimulate growth factor production. The double RNAi with either *bun* or *Madm* led to suppression of proliferation (Fig. 2f).

These results together demonstrate that the Bun and Madm requirement for ISC proliferation is epistatic to all the regulatory pathways tested. Therefore, we speculate that Bun and Madm do not function as specific components downstream of one of these signaling pathways but instead function to maintain an intrinsic ISC property that is needed for proliferation.

### BunA Localizes in Cytoplasm and Acts Synergistically with Madm to Promote ISC Proliferation

**Fig. 3:**
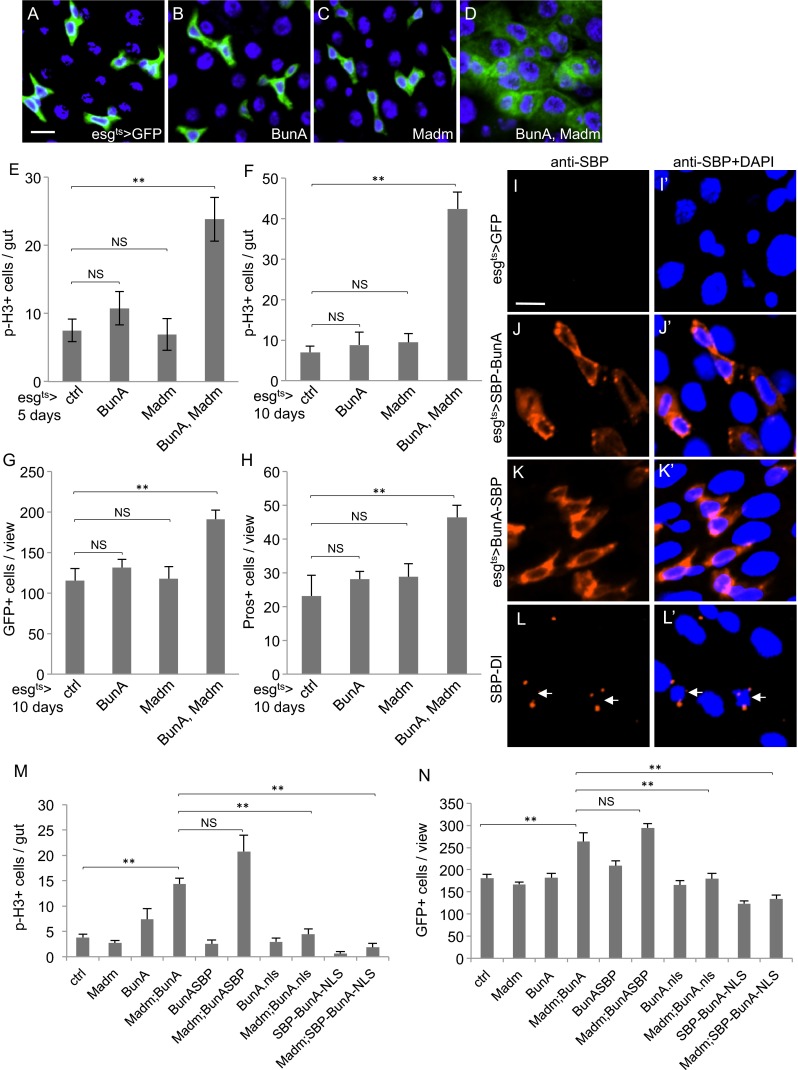
Bun localizes in cytoplasm and acts synergistically with Madm to promote ISC proliferation. **a-d** Confocal images of midguts from flies that expressed the transgenic UAS-BunA or UAS-Madm or the combination driven by the esg^ts^>. The shift to 29 °C was carried out for 5 days. All the fly strains also contain the UAS-GFP, represented by the green fluorescent signal of the images. DAPI staining for DNA is blue. **e-h** The same fly strains were used as in panel A-D. Panel E were from flies incubated at 29 °C for 5 days, and panel F-H were from flies incubated at 29 °C for 10 days. The p-H3+ staining was counted through out the midgut, and the GFP+ and Pros + cells were counted after taking microscopic images under the 40X objective. The average was plotted and more than 10 guts or images were counted for each sample. **i-l** Confocal images of midguts from flies that expressed the transgenic UAS-BunA tagged with SBP at N- or C-terminus. The antibody for immunofluorescent staining was directed against SBP. The expression was driven by esg^ts^ > and the flies were shifted to 29 °C for 24 h. The SBP-Delta (Dl) transgenic line expressed a fusion of SBP with an approximately 85 kb genomic *Delta* fragment containing the *Delta* promoter. **m-n** Quantification of p-H3+ staining and GFP+ cells after expressing the various combinations of transgenic UAS constructs as indicated. The BunA-nls construct contained one copy of SV40 nls and no SBP tag. The SBP-BunA-NLS construct contained multiple copies of sequences as SBP-NLS-BunA-2XNLS-SBP

We performed a series of experiments to investigate the function of Bun in the adult midgut precursors. Overexpression of BunA or Madm individually in the adult midgut did not affect proliferation (Fig. 3a-f). However, when both constructs were expressed, the p-H3 count in the midgut showed a significant increase either after 5 days (Fig. 3e) or 10 days (Fig. 3f). Quantification of the GFP+ precursor cells and Pros + EEs also showed consistent increase of midgut cells after the co-expression (Fig. 3g, h). These results are similar to that shown in eye development [36], and support the idea that BunA and Madm can function together in the adult midgut to promote ISC division.

We also investigated the subcellular localization by using the streptavidin binding protein (SBP) fusion with BunA at the N- or C-terminus. The proteins were expressed by using the temperature controlled esg^ts^ > system. Co-expression of these SBP-BunA fusion proteins with Madm in midguts increased proliferation, similar to that of the wild type BunA construct (Fig. 3m, n), indicating the SBP tag does not change the functionality of the protein. By immunofluorescence staining, the SBP antibody could already detect the fusion protein signal as early as 6 h after temperature shift to 29 °C, and in comparison no signal was present in control guts (Fig. 3i-k). The signal increased continuously and approached saturation after approximately 24 h at 29 °C. Both low and high level of expression of either constructs resulted in predominantly cytoplasmic staining, and some punctate patterns especially at earlier time points (Fig. 3j, k). The expression of an SBP-Delta fusion protein under the Delta genomic promoter gave a punctate SBP staining that was similar to Delta antibody staining in ISCs (Fig. 3l), suggesting that the SBP fusion does not change the protein localization. Previous transfection experiments in S2 cells and antibody staining for endogenous Bun protein in eye imaginal discs also suggested a cytoplasmic localization of Bun [36, 44], consistent with our observation here.

To investigate further the function of BunA, we examined an untagged BunA construct containing a nuclear localization signal (nls) (Fig. S3C). Co-expression of this BunA-nls with Madm resulted in a weaker activation of midgut proliferation when compared to that of wild type BunA with Madm (Fig. 3m, n). Furthermore, we examined a few SBP-tagged BunA constructs with varying copies of NLS (we use this notation to distinguish from the untagged nls version). A transgenic construct containing the SBP and NLS at both ends (SBP-NLS-BunA-2XNLS-SBP) showed detectable albeit weak SBP staining in the nuclei of midgut precursor cells when expressed by the esg^ts^ > GFP driver (Fig. S3D-E). This construct also showed no increase of proliferation when co-expressed with Madm (Fig. 3m, n). While there could be alternative interpretations of these results, we favor the idea that BunA normally functions in the cytoplasm and interacts with Madm to enhance ISC proliferation.

### Bun Acts Downstream of Tuberous Sclerosis Complex to Regulate Phosphorylated 4EBP and ISC Growth

Cell division requires many components that need to be coordinated at various phases of the cell cycle. One important aspect is cellular growth, which is essential to produce two cells that have the same size as the original cell before division [45]. Cell growth requires substantial protein synthesis and the TOR pathway plays an important role in integrating growth stimulatory signals with the translation machinery [46]. The TOR pathway is normally suppressed by the Tuberous Sclerosis Complex, which contains 2 proteins called Tuberous Sclerosis Complex 1 and 2. In the absence of either Tuberous Sclerosis Complex 1 or 2, the GTPase Rheb is activated to promote TOR to phosphorylate 4EBP, which in turn is released from and allows eIF4E to promote translation.

**Fig. 4:**
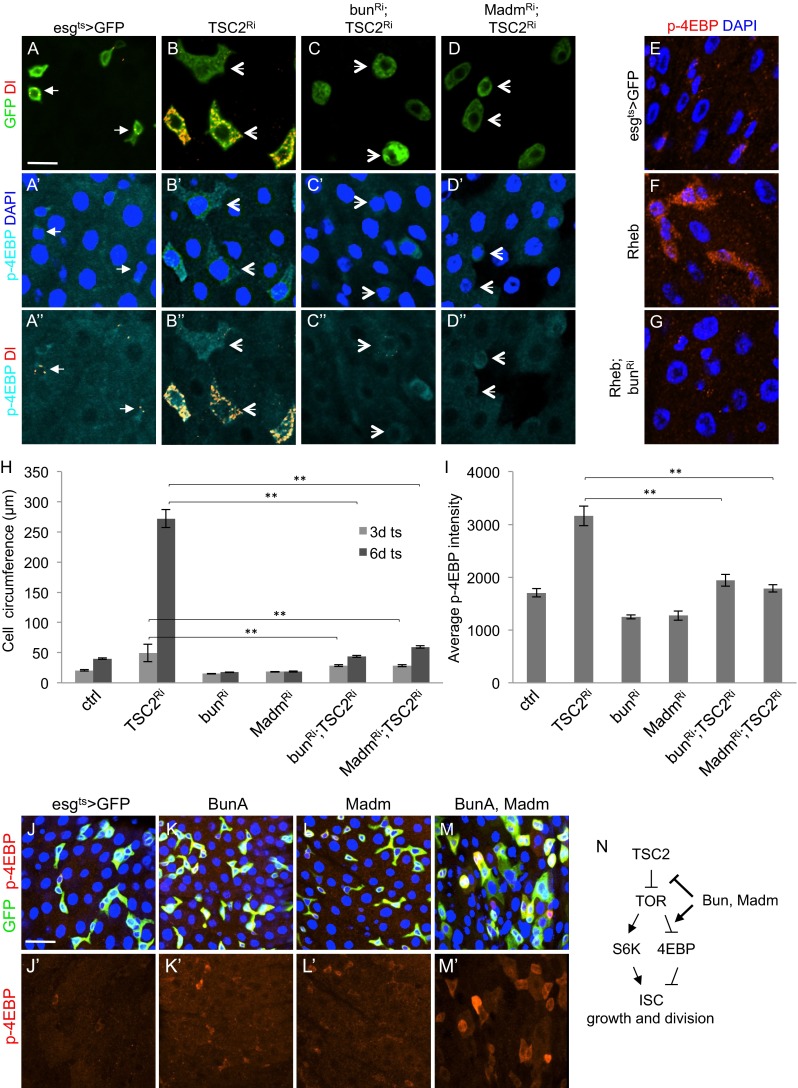
Bun acts downstream of Tuberous Sclerosis Complex to regulate 4EBP and ISC growth. **a-d** Confocal images of midguts from adults flies with the esg^ts^ > RNAi constructs as indicated. All crosses also contained the UAS-GFP. Pupae were reared at 29 °C from 2 days before adult fly eclosion and continued for 4 more days after eclosion; this condition gives the best cell growth after *TSC2* RNAi. Midguts were dissected and used for anti-phospho-4EBP (p-4EBP) and anti-Delta (Dl) staining. **e-g** Confocal images showing p-4EBP staining of midguts from flies containing the UAS-GFP control, UAS-Rheb overexpression construct, or together with the *bun* ^*RNAi*^ as indicated. **h-i** The circumference of GFP+ cells in the confocal images was measured and the average was plotted. More than 30 cells were measured for each genotype. For the same cells, the p-4EBP staining signal was also quantified as fluorescent intensity in each GFP+ cell. The signal was averaged and plotted as shown. **j-m** Confocal images showing p-4EBP staining of midguts from flies overexpressing BunA, Madm or the combination. **n** A model of Bun and Madm function in the TOR pathway regulating 4EBP phosphorylation and ISC growth, which is required for ISC division. Bun and Madm should function downstream of Tuberous Sclerosis Complex 2 (TSC2) and Rheb but the molecular mechanism remains to be determined

In the adult midgut, loss of function of Tuberous Sclerosis Complex leads to a highly increased ISC size, concomitant with a block of cell division although the Delta expression remains in these enlarged cells (Fig. 4a, b) [23]. The inclusion of *bun*^*RNAi*^ or *Madm*^*RNAi*^ constructs caused a substantial suppression of this cell size increase, as measured by the cell circumference in confocal images (Fig. 4c, d, h). Staining of wild type midguts by using an antibody for phosphorylated 4EBP (p-4EBP) usually yielded only weak, sporadic signals (Fig. 4a’). However, *Tuberous Sclerosis Complex 2*^*RNAi*^ caused the p-4EBP staining to become more prominent in the cytoplasm of the big cells that also contained Delta staining (Fig. 4b-b”, I), suggesting that these were over-grown ISCs. This p-4EBP staining was efficiently suppressed by the presence of the *bun*^*RNAi*^ or *Madm*^*RNAi*^ constructs (4C-C”, D-D”). The overexpression of the GTPase Rheb, another component of the TSC-TOR pathway, yielded a similar increase of p-4EBP staining, which was suppressed by *bun*^*RNAi*^ (Fig. 4e-g).

We also examined midguts with BunA and Madm overexpression. While neither one alone could increase the p-4EBP staining, co-expression of both caused a detectable p-4EBP staining (Fig. 4j-m). This is consistent with the results shown above that overexpression of one of these proteins could not increase ISC proliferation while co-expression of BunA and Madm could.

Previous reports that analysed the mutant phenotypes in imaginal discs and ovary follicle cells have shown that *bun* or *Madm* mutant cells do not have substantial apoptosis, while cell division and growth were reduced [27, 28, 36, 47]. In the midgut, we did not observe increased caspase 3 staining. Moreover, overexpression of the apoptosis inhibitor P35 did not cause substantial rescue of the phenotype (figure S3A, B). Our data suggest that cell death is probably not the primary pathway that Bun and Madm regulate. However, cell growth defects would eventually lead to some form of cell death, including apoptosis or autophagy, which may manifest as late phenotypes after loss of Bun or Madm.

## Discussion

In this report, we show that Bun and Madm are intrinsically required for ISC growth and division. Our results here together with previous papers suggest a model that Bun and Madm form a complex in the cytoplasm to promote cellular growth and proliferation. The evidence that support this model includes transgenic expressed Bun localizes in the cytoplasm of midgut precursor cells, similar to the results from transfection in S2 cells and immune-staining in eye discs [27, 44]. Bun physically and functionally interacts with Madm, which has also been proposed as a cytoplasmic adaptor protein [36]. Adding a nuclear localization signal to Bun reduced the growth promoting ability of Bun. Although there is a possibility this signal peptide changes the functionality in an unpredicted way, we favor the interpretation that Bun normally acts in the cytoplasm and with Madm to regulate the proliferation of ISCs. This is in contrast to mammalian TSC-22, which was reported to function in the nucleus [48].

Our results presented here seem to contradict a previous publication reporting that TSC-22 arrests proliferation during human colon epithelial cell differentiation [49]. However, this apparent contradiction is resolved when we consider the growing evidence for distinct functions for large and small Bun/TSC-22 isoforms. The Bun/TSC-22 proteins have short and long isoforms that contain the conserved TSC-box and leucine zippers in the C-terminal domain. The prototypical TSC-22 protein, TSC22D1-001, may act as a transcriptional regulator and repress cancer cell proliferation, particularly for blood lineages [31, 33–35, 50]. Another recent model suggests that in Drosophila the long Bun isoforms interact with Madm and have a growth promoting activity, which is inhibited by the short Bun isoforms [27, 28, 36]. Similarly, the long isoform, TSC22D1-002, enhances proliferation in mouse mammary glands, whereas the short isoform promotes apoptosis [51]. Our unpublished result of transgenic expression of BunB also has lower function than BunA in fly intestinal progenitor cells is consistent with this model where large isoforms have a distinct function, namely in growth promotion.

Loss of either Bun or Madm can potently suppress all the growth stimulation by multiple pathways in the midgut as shown in this report. We interpret these results to indicate that Bun and Madm do not act specifically in one of the signaling pathways we tested but instead function in a fundamental process required for cell growth, such as protein synthesis or protein turnover. We therefore speculated that Bun and Madm may regulate the TOR pathway. In support of this idea, we show that *bun*^*RNAi*^ or *Madm*^*RNAi*^ efficiently suppresses the *Tuberous Sclerosis Complex 2*^*RNAi*^-induced cell growth and p4EBP phenotypes. A recent study of genetic suppression of TOR complex 1-S6K function in S2 cells also suggests that Bun and Madm can interact with this pathway [52]. Furthermore, proteomic analyses of Bun and Madm interacting proteins in S2 cells have shown interactions with ribosomal proteins and translation initiation factors [36]. Therefore, we propose a model (Fig. 4n) that Bun and Madm function in the Tuberous Sclerosis Complex-TOR-4EBP pathway to regulate protein synthesis in ISCs for their growth, which is a prerequisite for ISC proliferation. Suppression of Tuberous Sclerosis Complex mutant cell growth phenotype by *bun* or *Madm* RNAi was substantial but not complete (see Fig. 4). Earlier papers demonstrated that Bun also interacts with Notch and EGF pathway in ovary follicle cells [47, 53]. Therefore by definition Bun and Madm are neither 100 % essential nor restricted to the TOR pathway. Our genetic data suggest that Bun and Madm work downstream of Tuberous Sclerosis Complex and upstream of 4EBP, but they could also work in parallel to the TOR pathway components.

ISCs with loss of Tuberous Sclerosis Complex function have substantial cell size increase [23]. Meanwhile, the Bun/Madm overexpression caused increased ISC division but not cell hypertrophy. Both loss of Tuberous Sclerosis Complex and overexpression of Bun/Madm should promote cell growth but the phenotypes at the end are different. We speculate that the reason is the Bun/Madm overexpressing ISCs are still capable of mitosis, while the Tuberous Sclerosis Complex mutant ISCs do not divide anymore thereby resulting in the very big cells [23]. In Bun and Madm overexpressing midguts, the p-H3+ and GFP+ cell count showed a significant increase, indicating increased mitosis. Therefore, an explanation is that Bun and Madm overexpression may increase cell size/cell growth, but when they grow to certain size they divide, resulting in rather normal cell size.

The knockout of the Madm mammalian homolog, NRBP1, can cause accumulation of the short isoform TSC22D2 [54]. Up-regulation of Madm/NRBP1 has been associated with poor clinical outcome and increased growth of prostate cancer [55]. Further analysis based on our model may reveal whether high ratio of long Bun/TSC22 isoforms over short isoforms may associate with high Madm activity and poor clinical outcomes.

## Electronic supplementary material

Fig. S1 **A** Drosophila midgut ISC renewal and differentiation pathway. An ISC divides to make two daughter cells, one forms an ISC again and the other becomes an EB that goes into differentiation to become an EC or EE. esg > GFP marks both precursor cells, that is ISC and EB. Each of the cell types ISC, EB, EE and EC can be specifically marked by Delta, Notch pathway reporter Su(H)lacZ, Prospero and Pdm1, respectively. ISC: intestinal stem cell; EB: enteroblast; EC: enterocyte; EE: enteroendocrine cell. **B-D** *bun* ^*RNAi*^ or *Madm* ^*RNAi*^ did not change the ISC cell fate. Green is esg^ts^ > GFP, red is Delta staining, turquoise is Su(H)lacZ and blue is DAPI. Only one cell of the precursor doublets remained positively stained for Su(H)lacZ or Delta but not both, indicating no switching of cell fate. Panels B”’, C”’ and D”’ are enlarged views representing the respective boxed areas in the other panels. Arrows indicate wild type Delta + cells, and arrowheads indicate mutant RNAi cells. **E-F** Schematic diagram of the Bun and Madm proteins. The targeting sequences of the RNAi lines used in this study are as indicated to the top of each panel. (PDF 477 kb)

Fig. S2 Examination of knockdown efficiencies of the *bun* and *Madm* RNAi. **A-G** Assessment of *bun* RNAi by using the SBP-BunA protein expression and antibody staining. The UAS-SBP-BunA or BunA-SBP constructs were crossed together with the bunRNAi1 or 2 constructs. They were then crossed with the esg^ts^ > GFP driver line and the flies collected for temperature shift to 29 °C for 3 days to allow the expression of dsRNA and SBP-BunA fusion. The guts were then dissected and stained for protein expression using antibody for SBP. **H** Assessment of *Madm* RNAi by qPCR. The *Madm* dsRNA lines were crossed together with the tubulin^ts^ Gal4 driver. The flies were shifted to 29 °C for 3 days and the midguts were dissected for RNA isolation and qPCR analysis. The cross with *w-* was used as a control and the RNA level based on the PCR cycle number was set as 1. The PCR results of midguts from *Madm* RNAi flies were plotted as a fraction of the control. (PDF 361 kb)

Fig. S3 Analysis of cell death inhibition on *bun* and *Madm* RNAi phenotype and of Bun constructs containing the nuclear localization signal. **A-B** The *bun* and *Madm* RNAi lines were crossed together with the UAS-P35, encoding the insect anti-apoptotic protein. The expression was driven by the esg^ts^ > GFP driver. The flies were shifted to 29 °C for 5 days and midguts were dissected for p-H3 staining and quantification. Similar experiments were performed using the control and UAS-P35 transgenic flies after feeding with bleomycin, which is a DNA damaging agent that causes EC damage leading to ISC proliferation. The presence of P35 can suppress partially this damage induced ISC proliferation as shown in panel B. **C** The schematic representation of the untagged UAS-BunA-nls construct and the sequence. The transgenic flies were used for PCR of genomic DNA using the primers JCD69 and 70 as indicated. The PCR products were sequenced to confirm the presence of one copy of SV40 nls sequence. **D-E** Transgenic expression of SBP-tagged BunA and BunA-NLS constructs. The copy number of SV40 NLS and SBP are as indicated in panel E. The transgenic constructs were crossed with the esg^ts^ > GFP driver and midguts were dissected for staining using the anti-SBP antibody. Blue is DAPI staining for nuclear DNA. (PDF 815 kb)
