# Supplementary figures and images for: Bunched and Madm Function Downstream of Tuberous Sclerosis Complex to Regulate the Growth of Intestinal Stem Cells in Drosophila
Source: Stem Cell Rev. 2015 Sep 2;11(6):813–25. doi: 10.1007/s12015-015-9617-5 (PMC4653243; doi:10.1007/s12015-015-9617-5)

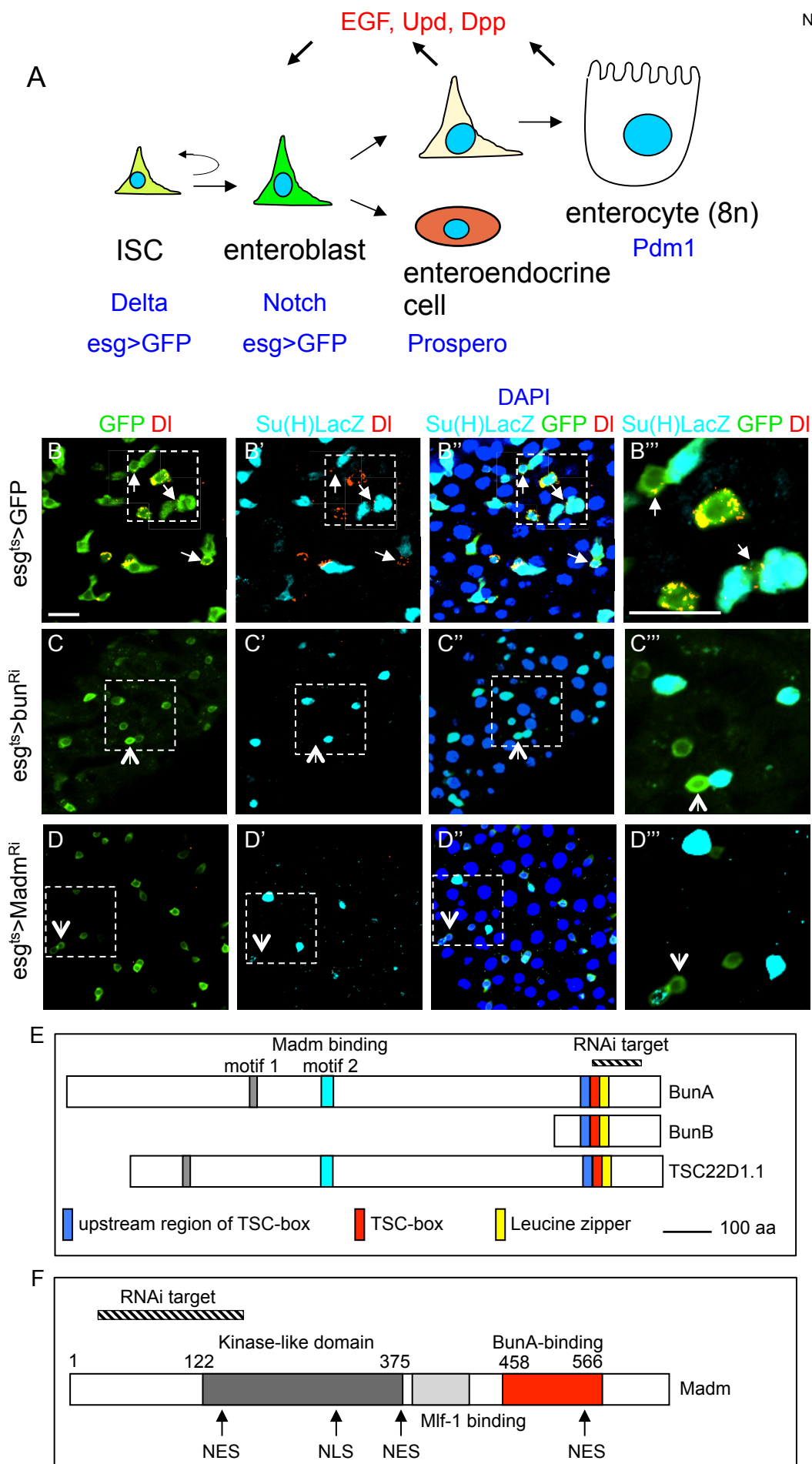

Supplement: Supplementary file 1 — A Drosophila midgut ISC renewal and differentiation pathway. An ISC divides to make two daughter cells, one forms an ISC again and the other becomes an EB that goes into differentiation to become an EC or EE. esg > GFP marks both precursor cells, that is ISC and EB. Each of the cell types ISC, EB, EE and EC can be specifically marked by Delta, Notch pathway reporter Su(H)lacZ, Prospero and Pdm1, respectively. ISC: intestinal stem cell; EB: enteroblast; EC: enterocyte; EE: enteroendocrine cell. B-D bun RNAi or Madm RNAi did not change the ISC cell fate. Green is esgts > GFP, red is Delta staining, turquoise is Su(H)lacZ and blue is DAPI. Only one cell of the precursor doublets remained positively stained for Su(H)lacZ or Delta but not both, indicating no switching of cell fate. Panels B”’, C”’ and D”’ are enlarged views representing the respective boxed areas in the other panels. Arrows indicate wild type Delta + cells, and arrowheads indicate mutant RNAi cells. E-F Schematic diagram of the Bun and Madm proteins. The targeting sequences of the RNAi lines used in this study are as indicated to the top of each panel. (PDF 477 kb) [file 12015_2015_9617_MOESM1_ESM.pdf]

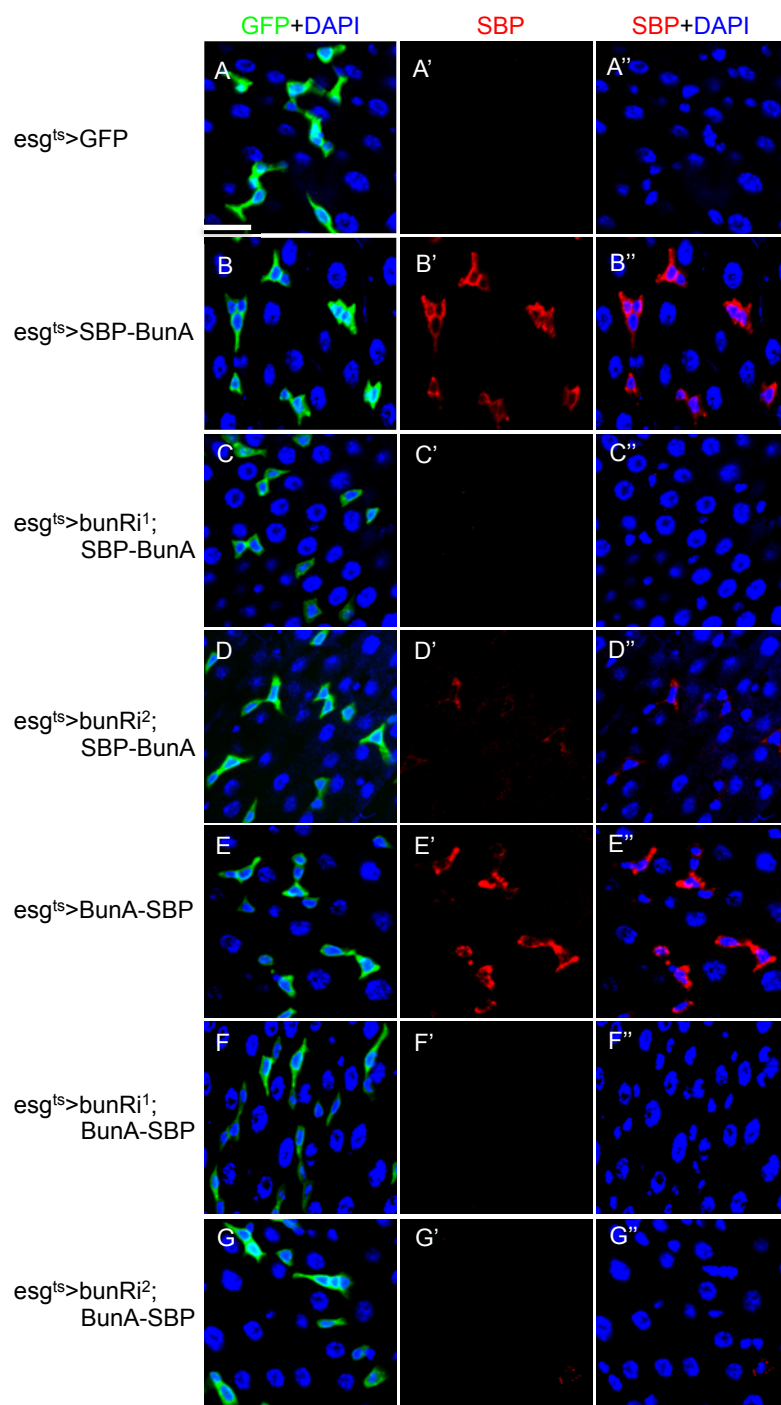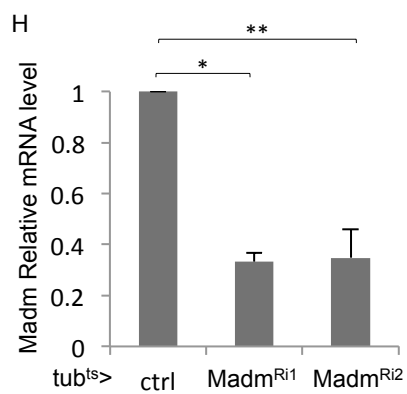

Supplement: Supplementary file 2 — Examination of knockdown efficiencies of the bun and Madm RNAi. A-G Assessment of bun RNAi by using the SBP-BunA protein expression and antibody staining. The UAS-SBP-BunA or BunA-SBP constructs were crossed together with the bunRNAi1 or 2 constructs. They were then crossed with the esgts > GFP driver line and the flies collected for temperature shift to 29 °C for 3 days to allow the expression of dsRNA and SBP-BunA fusion. The guts were then dissected and stained for protein expression using antibody for SBP. H Assessment of Madm RNAi by qPCR. The Madm dsRNA lines were crossed together with the tubulints Gal4 driver. The flies were shifted to 29 °C for 3 days and the midguts were dissected for RNA isolation and qPCR analysis. The cross with w- was used as a control and the RNA level based on the PCR cycle number was set as 1. The PCR results of midguts from Madm RNAi flies were plotted as a fraction of the control. (PDF 361 kb) [file 12015_2015_9617_MOESM2_ESM.pdf]

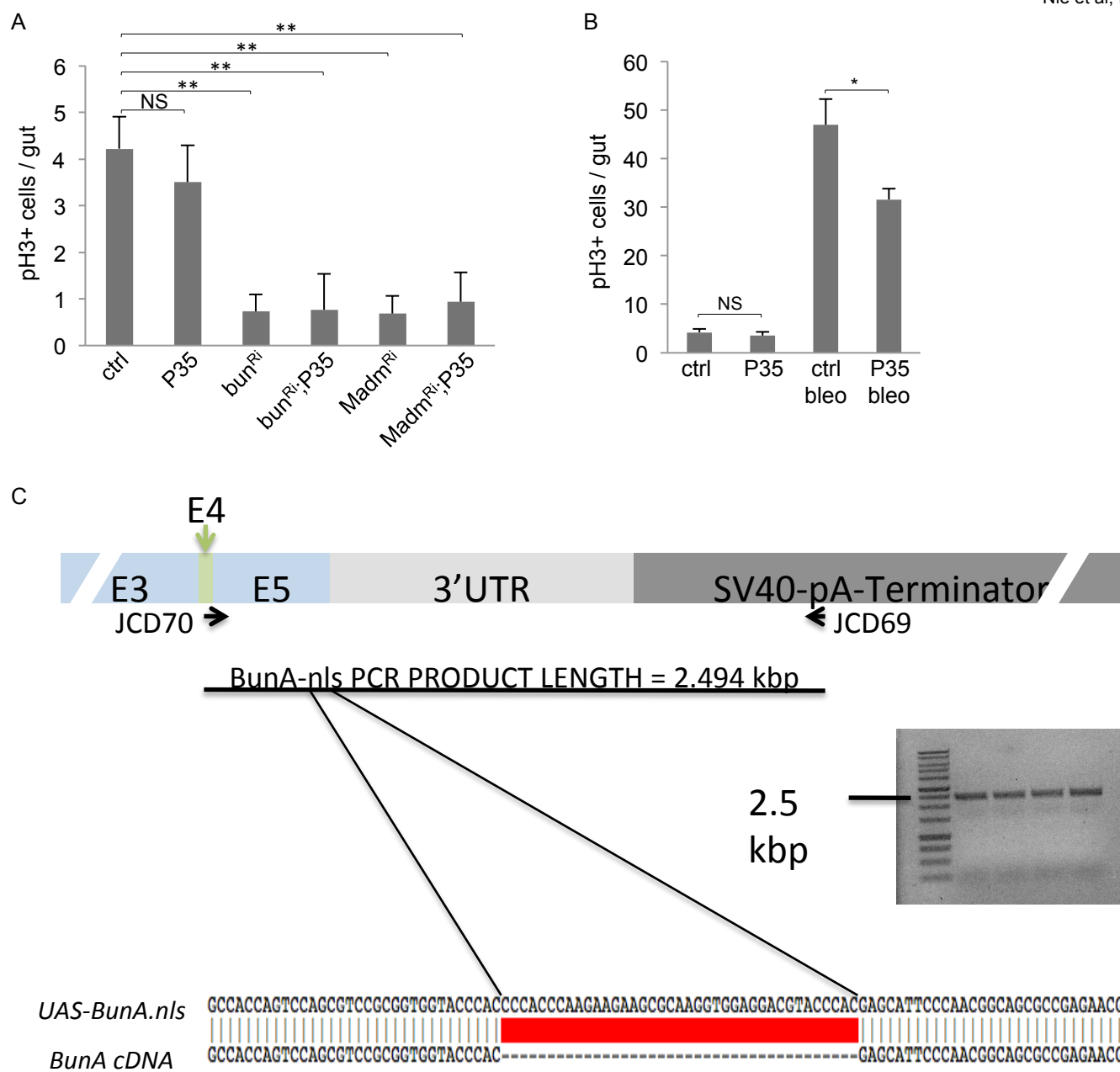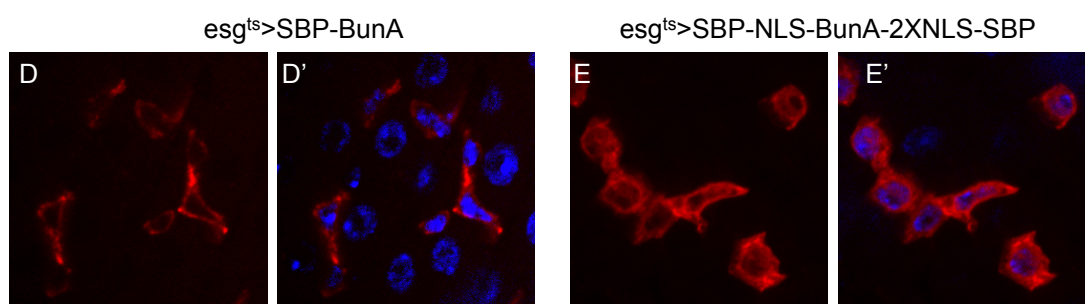

Supplement: Supplementary file 3 — Analysis of cell death inhibition on bun and Madm RNAi phenotype and of Bun constructs containing the nuclear localization signal. A-B The bun and Madm RNAi lines were crossed together with the UAS-P35, encoding the insect anti-apoptotic protein. The expression was driven by the esgts > GFP driver. The flies were shifted to 29 °C for 5 days and midguts were dissected for p-H3 staining and quantification. Similar experiments were performed using the control and UAS-P35 transgenic flies after feeding with bleomycin, which is a DNA damaging agent that causes EC damage leading to ISC proliferation. The presence of P35 can suppress partially this damage induced ISC proliferation as shown in panel B. C The schematic representation of the untagged UAS-BunA-nls construct and the sequence. The transgenic flies were used for PCR of genomic DNA using the primers JCD69 and 70 as indicated. The PCR products were sequenced to confirm the presence of one copy of SV40 nls sequence. D-E Transgenic expression of SBP-tagged BunA and BunA-NLS constructs. The copy number of SV40 NLS and SBP are as indicated in panel E. The transgenic constructs were crossed with the esgts > GFP driver and midguts were dissected for staining using the anti-SBP antibody. Blue is DAPI staining for nuclear DNA. (PDF 815 kb) [file 12015_2015_9617_MOESM3_ESM.pdf]
